# Lithotomy

**Published:** 1853-08

**Authors:** 


*Lithotomy.*—The extraction of a urinary calculus from the bladder by the recto-urethral operation has been now successfully accomplished by Mr. Lloyd, in three instances, at St. Bartholomew's Hospital. The stone was removed quickly, and the patients have done well. It may, therefore, be interesting to state, that the proceeding resembles, in great measure, the operation performed by Vacca Berlinghieri, and described by Malgaigne. (*Manuel de Médicine Opératoire*, p. 686, 1843.) The subject having been placed as in the lateral operation, a staff is introdued into the bladder, and given to an assistant, who is directed to hold it in a vertical direction, so that the groove should correspond exactly with the mesial line. The index finger of the left hand is introduced into the rectum, the palmar surface being turned forwards. Upon its flat surface a straight bistoury is inserted to a depth of eighteen millimetres (about nine lines) from the margin of the anus. The handle of the knife being then depressed, the point is thrust through the walls of the intestine, and the edge being also carried upwards, the sphincter ani, the inferior part of the rectum, the perinæum from the anus to the bulb, and the cellular interval which separates these parts, are divided together. The inferior region of the prostate can then be felt through the wound. In front is felt the membranous portion of the urethra containing the staff. The nail of the index finger is next used, as in the lateral operation, to discover the groove in the staff, and to conduct to it the point of the bistoury. The staff is then withdrawn, and the bistoury is thrust into the bladder; the edge is turned downwards, so as to divide partially, but not completely, the prostate in the mesial line, care being taken to avoid re-entering the incision of the rectum. Therefore, this operation effects the division of those parts which were torn in the old operation with which the "apparatus major" was associated. Mr. Lloyd introduces a speculum into the rectum; his incision is, perhaps, somewhat deeper than that of Vacca Berlinghieri; he *dilates*, but does not cut the prostate, unless the stone should be of very considerable size.

The incision appears remarkaby small when the operation is concluded; and, as Malgaigne has observed, at each effort of excretion, the mucous membrane descends over the wound, which is thus placed under conditions most favorable to healing without leaving a fistula.—*Med. Times and Gaz.*

